# Reemergence of yellow fever in Ethiopia after 50 years, 2013: epidemiological and entomological investigations

**DOI:** 10.1186/s12879-017-2435-4

**Published:** 2017-05-15

**Authors:** Abrham Lilay, Negga Asamene, Abyot Bekele, Mesfin Mengesha, Milliyon Wendabeku, Israel Tareke, Abiy Girmay, Yonas Wuletaw, Abate Adossa, Yamar Ba, Amadou Sall, Daddi Jima, Debritu Mengesha

**Affiliations:** 1 Ethiopian Public Health Institute-Ethiopia, PO Box: 1242, Dakar, Senegal; 2 WHO Country Office-Ethiopia, Dakar, Senegal; 3 Regional Health Bureau of the Southern Nations Nationalities and Peoples-Ethiopia, Dakar, Senegal; 4 WHO Collaborating Center for Arboviruses and Hemorrhage Fevers, Dakar, Senegal

**Keywords:** Yellow fever, Mosquito, Outbreak, Ethiopia

## Abstract

**Background:**

Yellow Fever (YF) is a viral hemorrhagic disease transmitted by *aedes mosquito* species. Approximately, 200,000 cases and 30,000 deaths occur worldwide every year. In Ethiopia, the last outbreak was reported in 1966 with 2200 cases and 450 deaths. A number of cases with deaths from unknown febrile illness reported from South Ari district starting from November 2012. This investigation was conducted to identify the causative agent, source of the outbreak and recommend appropriate interventions.

**Methods:**

Medical records were reviewed and Patients and clinicians involved in managing the case were interviewed. Descriptive data analysis was done by time, person and place. Serum samples were collected for serological analysis it was done using Enzyme-linked Immunosorbent Assay for initial screening and confirmatory tests were done using Plaque Reduction and Neutralization Test. Breteau and container indices were used for the entomological investigation to determine the risk of epidemic.

**Results:**

A total of 141 Suspected YF cases with 43 deaths (CFR = 30.5%) were reported from November 2012 to October 2013 from South Omo Zone. All age groups were affected (mean 27.5, Range 1–75 Years). Of the total cases, 85.1% cases had jaundice and 56.7% cases had fever. Seven of the 21 samples were IgM positive for YF virus. *Aedes bromeliae* and *Aedes aegypti* were identified as responsible vectors of YF in affected area. The Breteau indices of Arkisha and Aykamer Kebeles were 44.4% and 33.3%, whereas the container indices were 12.9% and 22.2%, respectively.

**Conclusion:**

The investigation revealed that YF outbreak was reemerged after 50 years in Ethiopia. Vaccination should be given for the affected and neighboring districts and Case based surveillance should be initiated to detect every case.

## Background

Yellow Fever (YF) is an acute and often fatal infectious disease caused by the YF virus (YFV), a flavivirus transmitted in tropical and subtropical areas, mainly through the bite of infected *Aedes spp.* mosquitoes in Africa, and by *Haemagogus spp.* mosquitoes in South America [1, 2]. It is characterized by acute onset of fever, chills, headache, backache, generalized muscle pain, nausea and vomiting. The clinical presentation ranges from asymptomatic to classical hemorrhagic fever and death. In most instances the clinical manifestation follows three phases; acute, remission and toxic phases. Most cases improve and recover within 4 to 5 days. Some cases will undergo temporary remission phase for 24–48 h in which patients started to relive from the symptoms. However; about 15% to 25% of the cases enter into a toxic phase after 1 to 2 days of initial recovery [3].

YF is endemic in tropical areas of Africa and South America. Approximately 200,000 cases and 30, 000 deaths attributed to YF occur worldwide every year [2, 4, 5]; the majority of the deaths occur in the 33 sub-Saharan endemic countries where more than 500 million people are exposed [6, 7]. About 90% of the estimated cases were from Africa. A dramatic resurgence of YF has occurred since the 1980s in both sub-Saharan Africa and South America [3]. The Epidemiological and serological studies indicate that East Africa countries mainly Ethiopia (1960–62,1964), Kenya (1992–1993), Sudan (2003, 2005), South Sudan (2012) and Uganda (2010–2011) had affected by YF outbreaks [1, 5, 8–14].

Ethiopia is one of the YF transmission at risk countries in Africa [15, 16]. Different research and outbreak investigation reports showed that YFV had been circulating In Ethiopia [8, 17–19]. From 1960 to 1962, there was a large outbreak of YF in different parts Ethiopia mainly around Gamo Gofa, Jinka, Kaffa and Wollega areas which approximately affected about 100,000 persons and killed 30,000 (CFR: 30%). In this outbreak adults were slightly more frequently affected than children while men were more affected than women [14]. Similarly, in 1966, YF reappeared in Arba-Minch, east of Lake Abaya, in an area unaffected by the outbreak of 1960 and affected 2200 persons with 450 deaths [8] .

South Omo Zonal Health Department (ZHD) reported deaths with unknown causes from South Ari district to Public Health Emergency Management (PHEM) of the Southern Nations Nationalities and Peoples Region (SNNPR) in November 2012. The main clinical manifestations were fever, headache, nausea and bloody vomiting. The regional PHEM conducted field investigations in January and February 2013 but the cause of the outbreak could not be determined and the regional PHEM notified Ethiopian Public Health Institute (EPHI) in late of March 2013 for conducting further investigation and characterizing the outbreak. After the request, team from EPHI and World Health Organization (WHO) Country Office deployed to the affected area in the end of March 2013 to identify the causative agent, source of the outbreak and recommend appropriate interventions.

## Methods

Medical records were reviewed. Direct interview was conducted with patients and health workers from South Ari district. Cases were defined as any person with acute onset of fever followed by jaundice within two weeks of onset of first symptoms. Active case search was conducted house to house using a case definition. All identified cases were recorded on line list and descriptive data analysis was done by time, person and place using Microsoft Excel Sheet. The distribution of cases and deaths were depicted on map using arc Geographic Information System (GIS) 9.2. Twenty one serum samples were collected from patients having clinical manifestations typical of the outbreak and transported to virology laboratory at EPHI using liquid nitrogen. Two ELISA protocols were employed in the diagnosis of the initial samples; one was a CDC protocol for the diagnosis of IgM antibody against YFV and then we did another ELISA using the in house protocol from Institute Pastuer Dakar for differential diagnosis and to rule out other arboviruses. The results we found were using these two ELISA protocols were the same. For confirmation of the ELISA results, PRNT was performed at the reference laboratory. The Result was interpreted based on the kit insert and WHO Protocol. The household of each identified suspect case and its vicinity have been investigated for possible mosquito vectors. Artificial and natural mosquitoes breeding sites were inspected. As only the aquatic habitats harboring at least one larva or pupae of *aedes spp.* were considered positive, samples were collected from infested containers and returned to the laboratory (in sectary) for rearing and identification of the emerging adult stages. Breteau (i.e. total number of containers with larvae or pupae per 100 habitations units) and container (i.e. percentage (%) number of positive containers divided by the number of containers inspected) index risks were estimated with collected data. According to the WHO, there is an epidemic risk if the value of Breteau and container indexes is higher than 5% and 3% respectively [20]. For adult stages sampling; a back pack aspirator was used in indoors and outdoors of human dwellings but also in the peri-domestic vegetation. Spray catch using crude pyrethrum was also used to collect indoor resting adults. Mosquitoes were collected manually and labeled by place of location and date of collection and identified using mosquito identification key [21]. Due to time shortage and sites are hard to reach, only six Kebeles (lowest administrative unit in Ethiopia): Arkisha and Tenadam from Jinka town; Aykamer and Geza from South Ari district, Key Afer from Bena-Tsemay district and Hana from Selamago district were selected for the entomological investigation.

As a public health response, this investigation was not categorized as research and informed consent was not required. Serum samples were collected only aiming to investigate the underlying causative agent and to guide appropriate outbreak response. The direction was given from EPHI, the government organization which has a full mandate to conduct epidemiological investigation and respond to any public health emergencies.

## Results

**Fig. 1:**
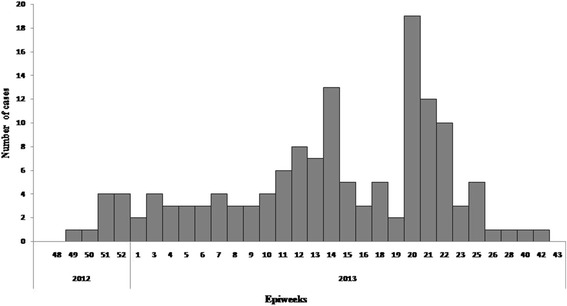
Distribution of Yellow Fever cases by epidemiological weeks – South Omo Zone, November 2012 – October 2013

A total of 141 (Mean 27.5 and Range:1–75 years) YF suspected and confirmed cases with 43 deaths (CFR = 30.5%) consistent with clinical manifestations of the cases reported were identified from November 2012 to October 2013 from South Ari, Bena-Tsemay, Jinka and Selamago districts of South Omo Zone. Of the total cases, 120 (85.1%) cases had jaundice, 80(56.7%) had fever, 61(43.3%) had bloody vomiting, 60 (42.6%) had nausea and 34 (24.1%) had headache (Fig. 1).

Considering the risk population of the affected districts, the crude attack rate (AR) was 0.04 and the sex specific AR was 0.03 and 0.05 in males and females, respectively. The AR among 15–44 years was 0.07 and 0.04 among 45 years and above, where as the AR among 0–5 and 5–14 years was 0.01 (Table 1). South Ari, Bena-Tsemay, Jinka and Selamago districts of South Omo Zone were affected by the outbreak, where majority of the cases, 106 (75.2%) were reported only from South Ari district.

**Table 1:** Attack rates of suspected Yellow Fever cases by Age group and Sex - South Omo Zone, November 2012–October 2013

Age group	Number of cases				Crude AR/100	Sex specific AR		Death	CFR
	Total	Percent	Male	Female		Male	Female		
0–4	3	2.1	0	3	0.01	0.00	0.01	0	0.0
5–14	15	10.6	8	7	0.01	0.02	0.01	2	13.3
15–44	106	75.2	44	62	0.07	0.06	0.08	36	34.0
≥45	17	12.1	8	9	0.04	0.04	0.04	5	29.4
Total	141	100.0	60	81	0.04	0.03	0.05	43	30.5

**Fig. 2:**
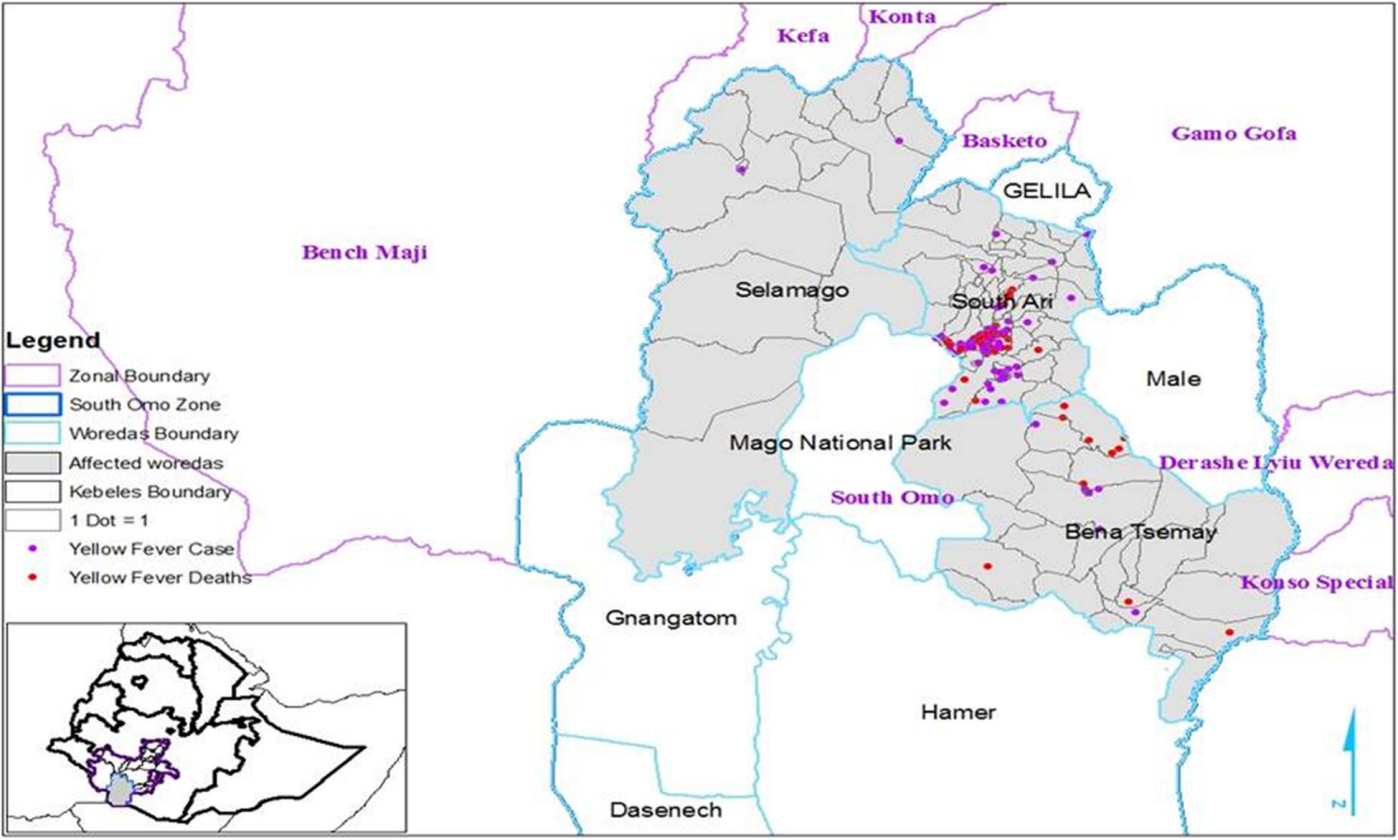
Distribution of cases and deaths of Yellow Fever in affected Kebeles by district - South Omo Zone, November 2012 – October 2013

Seven samples (33.3%) of the 21 serum samples collected were found IgM positive for YFV. On the other hand all samples tested using Real time PCR were negative for other arboviruses using In-House ELISA protocol for IgM detection from Institute Pasteur of Dakar, which is WHO reference laboratory for Arbo viruses and hemorrhagic fever viruses (Dengue fever, West Nile, Zika viruses, Rift Valley Fever, Crimean-Congo Hemorrhagic Fever and Chikungunya). Of the confirmed cases, four were from Aykamer Kebele of South Ari district. One confirmed case, each was found from Geza and Shepi Kebeles of South Ari district and Key Afer of Bena-Tsemay district (Fig. 2).

### Entomological investigation

A total of 87 natural and 67 artificial containers were found full of larvae. The natural breeding sites were mainly axil leaves of *Ensete ventricosum*, *Colocasia esculentum* plants while the artificial breeding places were usually found indoor and were mainly plastic containers used for water storage. The outdoor artificial breeding sites i.e. discarded containers which usually flooded by rains were often found dry as the investigation occurred at the end of the dry season (Table 2).

**Table 2:** Mosquito species and number of mosquitoes collected in affected Kebeles by spraying using mosquito killers – South Omo Zone, July 2013

Species	Arkisha		Tenadam		Aykamer		Geza		Hana		Key Afer		Total
	Spray	Asp	Spray	Asp	Spray	Asp	Spray	Asp	Spray	Asp	Spray	Asp	
*Cx pipiens*	38	15	34	8	1	2	0	0	0	658	164	173	1093
*Cx decens*	-	1	-	-	-	-	-	-	-	2	-	-	3
*Cx perfuscus*	15	-	-	-	-	-	-	3	-	-	-	-	18
*Cx antennatus*	1	-	-	-	-	-	-	-	-	2	-	-	3
*Cx neavei*		-	-	-	-	-	-	-	-	2	-	-	2
*Cx duttoni*		-	-	-	-	-	-	-	-	-	-	6	6
*An gambiae*	3	-	-	-	-	-	-	-	-	-	-	-	3
*Ae bromeliae*	1	-	-		-	3	-	-	-	1	-	6	11
*Ae africanus*		-	-		-	5	-	-	-	-	-	-	5
*Ae aegypti*	2	-	-	-	-	-	-	-	-	-	-	-	2

A total of 1146 mosquitoes belonging to 10 species and three genera were collected, sorted and classified into 83 monospecific pools (Table 3). *Aedes aegypti formosu*s, *Aedes bromeliae* and *Aedes africanus* were the three YF vectors identified in adult stage. *Aedes aegypti* was collected in Arkisha, *Aedes africanus* in Jinka and *Aedes bromeliae* in Arkisha, Aykamer, Bena-Tsemay and plantations in Selamago. The BI was 12.90%, 22.22% and 6.25% for Arkisha, Tenadam and Key Afer Kebeles, respectively. Whereas, the CI was 44.44%, 33.33% and 33.33% in each Kebele mentioned earlier, respectively.

**Table 3:** Breteau and Container Indices in affected Kebeles – South Omo Zone, July 2013

District	Kebeles	Container Index (%)	Breteau Index (%)
Jinka	Arkisha	12.90	44.44
Jinka	Tenadam	0.00	0.00
South Ari	Aykamer	22.22	33.33
South Ari	Geza	0.00	0.00
Bena-Tsemay	Key Afer	6.25	33.33
Selamago	Hana	0.00	0.00

## Discussion

We investigated the reemergence of YF outbreak in Ethiopia after 50 years. The last outbreak was reported in 1966 [8, 22]. A single confirmed case of YF is considered as an outbreak in Ethiopia [23]. The seven confirmed cases strongly support that outbreak was reemerged in South Omo Zone. Jinka town and its surrounding district, South Ari, were the most affected area with incidence rate of 5.2 and 4.7 cases per/10, 000 population. This area is also affected in the 1960–1962 outbreaks [14]. The crude AR was 0.04 and the CFR was 30.5% during this outbreak. Compared to the other age groups, 15–44 years old were 75% of the patients with AR of 0.05% and females were relatively more affected with male to female ratio of 0.7:1. In the 1960–1962 YF epidemic in Ethiopia, the disease affected all age groups, with a male to female ratio of 1.6:1 having cumulative case fatality rate(CRF) of 30% [14]. The outbreak investigation in Northern Uganda showed that the cases were evenly distributed among all age groups (Mean 28.2, Range 3–83 Years (12). In Kenya, 81% of the patients with YF were less than 40 years of age and no cases were recorded in individuals less than 10 years of age while young male adults were at highest risk [13].

The local surveillance system did not timely detect and report the cases. This might be attributed to non specificity of case definition and unavailability of laboratory diagnostic system at the ground level. This also contributed to underreporting and makes the number of cases few. As a result, clinicians didn’t not suspected YF until the investigation team arrived in the area and conducted investigation. The investigation team was able to identify additional cases from patient medical records consistent with clinical manifestations of YF which were not previously reported to higher levels. On the other hand, only sever cases had visited health facilities to seek medical care, mild cases were not. The investigation team identified cases while conducting active case search from house to house in the Kebeles which were not able to visit health facilities due to many reasons.

The area is covered with different vegetations including *Ensete ventricosum* and *Colocasia Esculentum* which were found as main natural breeding sites. The ecological context with human settlements in the middle of the vegetation in area where the first YF cases were confirmed could suggest the reemergence of the virus from the local foci. Most of the cases were reported from rural Kebeles. The presence of *Aedes africanus* and *Aedes bromeliae* in the investigated households, their vicinity and the ecological context where confirmed cases were identified tend to suggest that YF sylvatic transmission reemerged with the involvement of *Aedes africanus* and was relayed by *Aedes bromeliae* in the urban and rural areas.

Investigation of immature stages of mosquitoes showed that the average number of larvae per breeding-place was higher in the natural than in the artificial breeding sites. The Breteau and container indices were above the threshold level in Key Afer, Aykamer and Arkisha Kebeles and suggest the presence of epidemic risks in these areas.

## Conclusion

The investigation uncovered that sylvatic YF outbreak was reemerged after 50 years in Ethiopia. Jinka and South Ari districts were the most affected areas. The outbreak affected all age groups with 30.5% CFR. The epidemic risk indices were over the risk threshold in Arkisha and Aykamer Kebeles. *Aedes aegypti* and *Aedes bromeliae* were the vectors responsible for reemergence of the outbreak. Vaccination need be given for affected and neighboring districts and of course for travelers traveling to the affected areas. Training should be given for health workers especially on YF case definition and on specimen collection and shipment to EPHI. Besides, YF case based surveillance should be initiated in the Zone.

## Abbreviations

AR: Attack Rate
CDF: Case Fatality Rate
ELISA: Enzyme Linked Immunosorbent Assay
EPHI: Ethiopian Public Health Institute
NA: Not applicable
PHEM: Public Health Emergency Management
PRNT: Plaque Reduction and Neutralization
WHO: World Health Organization
YFV: Yellow Fever virus
ZHD: South Omo Zonal Health Department
